# Features of the TCR repertoire associate with patients' clinical and molecular characteristics in acute myeloid leukemia

**DOI:** 10.3389/fimmu.2023.1236514

**Published:** 2023-10-19

**Authors:** Mateusz Pospiech, Mukund Tamizharasan, Yu-Chun Wei, Advaith Maya Sanjeev Kumar, Mimi Lou, Joshua Milstein, Houda Alachkar

**Affiliations:** ^1^ Department of Clinical Pharmacy, School of Pharmacy, University of Southern California, Los Angeles, CA, United States; ^2^ Department of Computer Science, University of Southern California, Los Angeles, CA, United States; ^3^ Department of Population and Public Health Sciences, Keck School of Medicine, University of Southern California, Los Angeles, CA, United States; ^4^ Norris Comprehensive Cancer Center, University of Southern California, Los Angeles, CA, United States

**Keywords:** acute myeloid leukemia, T cell receptor (TCR), WT1, RNA sequencing (RNA-Seq), TCGA

## Abstract

**Background:**

Allogeneic hematopoietic stem cell transplant remains the most effective strategy for patients with high-risk acute myeloid leukemia (AML). Leukemia-specific neoantigens presented by the major histocompatibility complexes (MHCs) are recognized by the T cell receptors (TCR) triggering the graft-versus-leukemia effect. A unique TCR signature is generated by a complex V(D)J rearrangement process to form TCR capable of binding to the peptide-MHC. The generated TCR repertoire undergoes dynamic changes with disease progression and treatment.

**Method:**

Here we applied two different computational tools (TRUST4 and MIXCR) to extract the TCR sequences from RNA-seq data from The Cancer Genome Atlas (TCGA) and examine the association between features of the TCR repertoire in adult patients with AML and their clinical and molecular characteristics.

**Results:**

We found that only ~30% of identified TCR CDR3s were shared by the two computational tools. Yet, patterns of TCR associations with patients’ clinical and molecular characteristics based on data obtained from either tool were similar. The numbers of unique TCR clones were highly correlated with patients’ white blood cell counts, bone marrow blast percentage, and peripheral blood blast percentage. Multivariable regressions of TCRA and TCRB median normalized number of unique clones with mutational status of AML patients using TRUST4 showed significant association of TCRA or TCRB with WT1 mutations, WBC count, %BM blast, and sex (adjusted in TCRB model). We observed a correlation between TCRA/B number of unique clones and the expression of T cells inhibitory signal genes (*TIGIT, LAG3*, *CTLA-4*) and *foxp3*, but not *IL2RA, CD69* and *TNFRSF9* suggestive of exhausted T cell phenotypes in AML.

**Conclusion:**

Benchmarking of computational tools is needed to increase the accuracy of the identified clones. The utilization of RNA-seq data enables identification of highly abundant TCRs and correlating these clones with patients’ clinical and molecular characteristics. This study further supports the value of high-resolution TCR-Seq analyses to characterize the TCR repertoire in patients.

## Introduction

Acute myeloid leukemia (AML) is a life-threatening hematologic malignancy characterized by the accumulation of highly proliferative and poorly differentiated myeloid cells in the blood and the bone marrow (1). It commonly affects older people with a median age at diagnosis of 68 years and a five year overall survival rate lower than 30% (1). Allogeneic hematopoietic stem cell transplant (allo-HSCT) remains the most effective therapeutic strategy for high-risk AML patients likely due to the graft versus leukemia (GvL) effect, in which donor T cells recognize and target leukemic clones (2, 3). In addition to driving disease development and progression, mutations in the leukemic cells (1, 4) present ideal leukemia specific neoantigens when presented by the major histocompatibility complexes (MHCs) and then recognized by the T cell receptors (TCR) triggering the GvL effect (1, 5). A unique TCR signature is generated by a complex rearrangement process of an array of variable (V), diversity (D), and joining (J) exons to select one of each that will recombine into a functional receptor with complementary determining regions 3 (CDR3) responsible for the interaction between the TCR and the peptide-MHC (6–8). The generated TCR repertoire undergoes dynamic changes with the onset and progression of disease and the course of treatment (9). Specific features of the TCR repertoire such as the TCR diversity and clonal expansion were found to correlate with a patients' clinical characteristics, disease status and clinical outcome (10–15).

In AML, an increase in TCR expanded clones following PD-1 blockade therapy was observed in responders’ patients (11). Furthermore, TCR diversity was lower in patients with graft versus host disease (GVHD) and in relapsed patients among those who received allo-HSCT (14). Diversity of the TCR repertoires undergoes significant skewing post-allo-HSCT with an increased clonal expansion compared with healthy subjects (16). Considering the clinical and biological relevance of the TCR repertoire, computational methods to extract the TCR sequences from widely available public RNA-seq data have increasingly become available (17, 18). Among these computational tools, TRUST was recently developed and applied to analyze public TCR/BCR repertoire from The Cancer Genome Atlas (TCGA) cancer data and in pediatric and adult AML (10, 19). Infants with AML had significantly higher TCR CDR3s per kilo TCR reads (CPK) compared with children or adult patients (10). The study also reported an association between CBFB-MYH11 mutation and lower TCRB CPK value in pediatric patients with AML (10).

Here we applied two computational methods (the updated version of TRUST (TRUST4) and MIXCR on the LAML-TCGA RNA-seq data to profile TCR repertoires across a cohort of 151 patients with AML to investigate the TCR repertoire in peripheral blood (PB) samples in relationship with patients’ clinical and molecular characteristics to better understand the role of TCR repertoire and its impact on disease progression and outcome. Our study serves as an independent unbiased approach to apply two commonly used computational tools to analyze TCR clones from RNA-seq dataset in AML. This research provides valuable insights into the identified TCR clones from RNA-seq data and calls for the need of refinement and optimization of computational methodologies in this evolving field.

## Materials and methods

### TCGA repository data

We downloaded the clinical data from AML samples of 151 patients with complete clinical and RNA-seq data of peripheral blood at the time of leukemia diagnosis from The Cancer Genome Atlas (TCGA) repository from cbioportal (4, 20). Patient molecular and RNA-seq data were downloaded from TCGA-LAML project on the National Cancer Institute Genomic Data Commons (NCI GDC, https://portal.gdc.cancer.gov) portal (21). The patients were diagnosed and treated following National Comprehensive Cancer Network (NCCN) guidelines as reported in the original publication (4). Patients' clinical characteristics including age at diagnosis, sex, prior treatment, white blood cell count (WBC), bone marrow (BM) blast percentage, PB blast percentage, transplant status, disease free survival (DFS) and overall survival (OS) were obtained and included in the analysis along with molecular characteristics such as karyotype, cytogenetics, molecular and cytogenetic risk. Molecular and cytogenetic risk classifications were assigned in accordance with NCCN guidelines (www.nccn.org). The most common AML somatic mutations: *DNMT3*, *FLT3*, *NPM1*, *TET2*, *RUNX1*, *IDH1*/2, *TP53*, *CEBPA*, *NRAS*, *WT1* were also evaluated.

### Reconstructing the TCR repertoire from RNA-seq

Extraction and alignment of the TCR repertoire from RNA-seq data were performed using the TRUST4 tool (22) and MIXCR 3.0.13 tool (23) using default parameters. TRUST4 aligns raw sequencing reads to the reference V, D, J and C genes of T cell receptors from the ImmunoGeneTics (IMGT) database (24). The algorithm performs *de novo* assembly by aligning the candidate read to existing contigs and builds an index for all k-mers and extends the seed to identify alignments. TRUST4 uses IMGT to determine CDR3 coordinates after determination of V and J genes, in the final stage of analysis.

MIXCR initially aligns raw sequencing reads to reference V, D, J and C genes of T- cell receptors from fastq files using default parameters to generate *.vdjca file. Clonotypes are built from alignments by extracting the clonal sequence specified by assembling features parameter which by default is CDR3. Only good quality nucleotides are utilized to build core clonotypes with each being characterized by clonal sequence and a number of records associated with this clonotype. Initially, MIXCR assembles alignments that only partially cover CDR3 region. If V and/or J segments are determined, but CDR3 edges lack nucleotides MIXCR extends command imputes those from the germline. Alignments are then assembled into clonotypes, and errors are corrected. Final output text files include corresponding alignments, nucleotide and amino acid sequences of the gene region, clone count, and clone fraction.

### Analysis of immune repertoire sequencing

VDJtools 1.2.1 (25) was used to analyze the sequencing data V segment usage and clonotype sharing (the default parameters were used). To generate heatmaps of V segment usage for TRUST4 and MIXCR, we have converted output files to VDJtools format and utilized CalcSegmentUsage functionality that calculates V and J segment usage with assigned frequency of associated reads for each V/J exons present in the sample. MIXCR output contained 447 clones that were matched to multiple T cell receptor alpha V (*TRAV*) segments and 830 with multiple T cell receptor beta V *(TRBV)* segments. To resolve this, we filtered multiple V gene assignments where only the gene with the highest score for a certain clone was kept. Clones with multiple V exons having equal scores have been filtered out. Further, we used the OverlapPair function to perform a comprehensive analysis of clonotype sharing between MIXCR and TRUST.

### Identification of antigen specific TCR repertoires

VDJdb (26) was used to determine target epitopes to which the candidate TCR is predicted to bind based on a database consisting of the results of published T-cell specificity assays coupling antigen specificities with TCR sequences.

### TCR clone clusters identification

To identify T cell receptor beta (TCRB) clusters characterized by high probability of sharing antigen specificity we applied GLIPH2 (grouping of lymphocyte interaction by paratope hotspots version 2) (27) to cluster TRUST4 and MIXCR TCRB clones from all patients. Significant clonal groups determined by GLIPH2 were selected based on local motif-based similarity. The confidence of determined clusters was evaluated by Fisher’s exact test, which checks for the enrichment of unique CDR3s in said cluster compared to the reference naive CD4^+^ and CD8^+^ as provided by GLIPH2 (27).

The sequence logos were generated in R using ggseqlogo (28). The relative size of each aa symbol is proportional to its frequency in the dataset, while the total height of aa symbols indicates the information content of the position in bits.

### Data analysis

Since the low reads corresponding to the TCR impact the accuracy of diversity estimates, such as Shannon Diversity Index or Simpson’s Index, which are commonly used in TCR repertoire analysis, we used a normalized unique clone count as an approximate metric of TCR diversity as described by previous study (10). The unique clone count represents the number of unique TCR-CDR3 sequences that are identified within a sample. To account for differences between samples based on variability in the number of the sequencing reads and differences in the T cell content we normalized the number of unique clones by dividing it by the total number of RNA-seq reads specific to each sample and additionally dividing by one minus PB blast percentage (10). This normalization ensures that samples with higher sequencing depth are not overrepresented in the analysis, which can introduce bias. Higher values of this metric suggest greater diversity of the TCR repertoire, while lower values suggest a more limited diversity. The exceptions are the analyses that included PB blast percentage as a variable, here the normalized numbers of unique clones were determined by dividing unique clone counts by the total number of RNA-seq reads specific for each sample without further normalization by the PB blasts percentage. Patients were excluded from all analyses when no TCR clones were detected for both T cell receptor alpha (TCRA) or TCRB or when missing full clinical or mutational data. Patients that did not have information about PB blast percentage at diagnosis were excluded from the normalized number of unique clone count analyses. The number of samples included for each analysis is included in the figure legends.

The Top 10 *TRAV* or *TRBV* genes were identified by VDJtools as the *TRAV* or *TRBV* genes with the highest average frequency within the cohort of patients with AML. We also defined the public CDR3 as CDR3 clones that are present in more than one patient.

### Statistical analysis

The TCRA/TCRB unique clone counts and normalized unique counts were compared according to patients' clinical and molecular characteristics using Kruskal Wallis with Dunn’s *post-hoc* (when comparing more than two groups) tests or parametric two-sample T test or ANOVA (when comparing more than two groups) were applied when the data followed a normal distribution tested by Shapiro-Wilk test. The type of the statistical test performed is provided in each of the figure legends. Overall survival was defined in the original TCGA study as the time between diagnosis and death due to any reason (4). Estimated probabilities of overall survival were calculated according to the Kaplan-Meier method and paired log-rank test evaluated differences between patients belonging to each quartile of the normalized unique TCRA and TCRB counts were evaluated using the paired log-rank test with Bonferroni correction where appropriate. Pearson correlation coefficients were calculated to assess associations between TCRA/TCRB and clinical and molecular characteristics. Statistical significance was determined on unadjusted p value with false discovery rate (FDR) correction which was performed by the Benjamini-Hochberg procedure for multiple comparisons and the q value within the text and figure legends was provided where appropriate. For the univariate and multivariable analyses, the log transformation was performed on the value of unique TCR clones normalized to the number of total sequencing reads. The statistical significance was considered when the p values both from MIXCR and TRUST4 were lower than 0.05 based on the intersection-union test. We identified a list of important variables via analyses of univariate regressions with p<0.2. The forward selection for the regression model building with Schwarz Information Criterion (SBC) was performed to select the significant variables for inclusion into the final regression model. The intersection-union principle was applied where appropriate. Data analyses were implemented in the Pandas 1.42 package in python or GraphPad Prism 9.0 and SAS v9.4. Figures were generated using GraphPad Prism 9.0, Seaborn package in python and VDJ tools.

## Results

### Estimation of TCR clones from RNA-seq TCGA data of patients with AML

**Figure 1:**
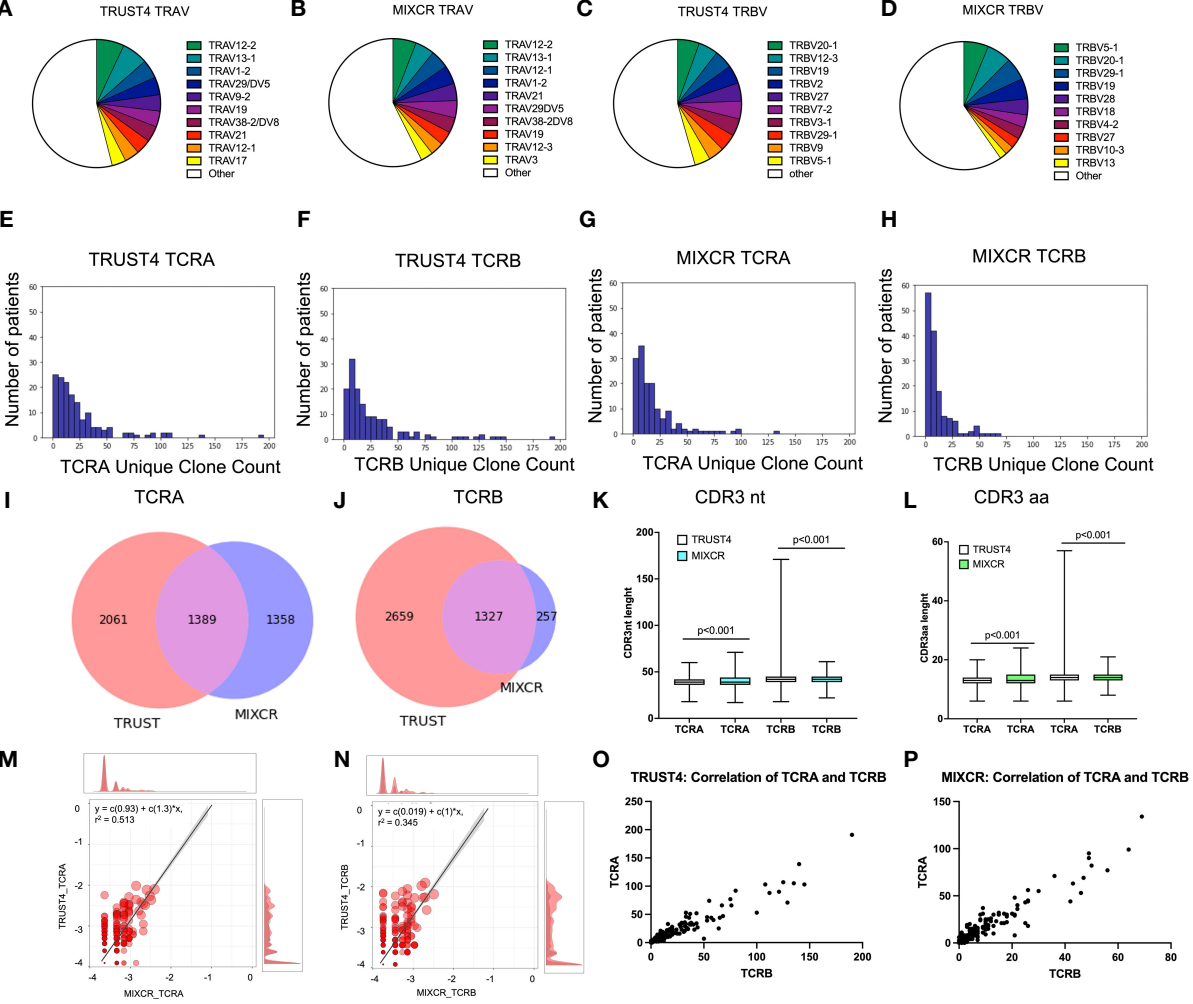
Comparison of identified clonotypes between MIXCR and TRUST4. *TRAV* segment usage by TRUST4 **(A)** and MIXCR **(B)**. *TRBV* segment usage by TRUST4 **(C)** and MIXCR **(D)**. Distribution of the unique TCRA clone **(E)** and TCRB **(F)** clone count using TRUST4. Distribution of the unique TCRA clone **(G)** and TCRB clone **(H)** count using MIXCR. Overlap of identified TCRA **(I)** and TCRB **(J)** CDR3s between MIXCR and TRUST4. CDR3nt **(K)** and CDR3aa **(L)** length comparison between TRUST4 and MIXCR. Scatterplot of top 20 TCRA **(M)** and TCRB **(N)** clonotype overlap between MIXCR and TRUST. Main graph consists of a scatterplot of overlapping clonotypes abundances and their linear regression. Each point represents the geometric mean of the clonotype frequency in both samples. Both axes are representing log10 of clonotype frequencies in each of the samples. Marginal histograms show the overlapping (red) and total (gray) clonotype abundance distributions weighted by clonotype abundance. Correlation between TCRA and TCRB in TRUST4 **(O)** and MIXCR **(P)**.

We analyzed the RNA-seq data from the TCGA dataset using both TRUST4 and MIXCR to identify TCR clones in patients with AML. The observed total numbers of aligned TCR reads for all samples were 8093 and 8253 for TCRA and TCRB, respectively by TRUST4; and 3541 and 1968 for TCRA and TCRB, respectively by MIXCR (**Table 1**). On average 55.5 ± 78.2 reads were aligned to TCRA and 56.6 ± 77.0 to TCRB by TRUST4 and 23.9 ± 26.9 reads were aligned to TCRA and 13.3 ± 16.7 to TCRB by MIXCR, per patient (**Table 1**). The identified TCR clones included most of the functional V and J exons (45 *TRAV* and 53 *TRBV*, 56 *TRAJ* and 13 TRBJ using TRUST 4, **Figures S1A, B**; and 48 *TRAV* and 36 *TRBV*, 52 *TRAJ* and 14 *TRBJ*, **Figures S1C, D** using MIXCR) indicating a reasonable RNA-seq read coverage of TCR genes. The top 10 *TRAV* which accounted for 46.04% of the TCRA repertoire in TRUST4 (**Figure 1A**) and 42.61% in MIXCR (**Figure 1B**), shared the following: *TRAV12-2*, *TRAV13-1*, *TRAV1-2*, *TRAV29DV5*, *TRAV19*, *TRAV38-2DV8*, *TRAV21*, and *TRAV12-1*. The Top 10 *TRBV* genes accounting for 45.56% of TCRB repertoire in TRUST4 (**Figure 1C**) and 40.28% in MIXCR (**Figure 1D**) shared the following five *TRBV* between TRUST4 and MIXCR: *TRBV20-1*, *TRBV19*, *TRBV27*, *TRBV29-1*, and *TRBV5-1*.

**Table 1:** Comparison of MIXCR and TRUST4 analysis output.

	TRUST4	MIXCR
Total number of TCR reads TCRA TCRB	8093 8253	3541 1968
Total number of reads per patient TCRA (M ± SD, range) TCRB (M ± SD, range)	55.5 ± 78.2, 1-506 56.6 ± 77.0, 1-360	23.9 ± 26.9, 1-162 13.3 ± 16.7, 1-88
Reads per clone TCRA (M ± SD, range) TCRB (M ± SD, range)	2.2 ± 4.0, 1-78 2.0 ± 4.6, 1-102	1.2 ± 0.9, 1-18 1.2 ± 0.8, 1-11
Identified V segments *TRAV* *TRBV*	45 53	48 36
Number of unique clones per patient TCRA (M ± SD, range) TCRB (M ± SD, range)	24.8 ± 28.6, 0-191 27.4 ± 33.4, 0-190	18.9 ± 21.6, 0-134 10.8 ± 13.2, 0-69
CDR3nt length TCRA (M ± SD) TCRB (M ± SD)	38.9 ± 6.1 41.6 ± 5.6	40.3 ± 5.2 40.6 ± 4.6
CDR3aa length TCRA (M ± SD) TCRB	12.9 ± 2.0 13.9 ± 1.9	13.5 ± 1.8 13.5 ± 1.5
Frequency per clone TCRA (M ± SD, range) TCRB (M ± SD, range)	0.04 ± 0.06, 0.002-1 0.03 ± 0.07, 0.003-1	0.05 ± 0.08, 0.006-1 0.07 ± 0.13, 0.011-1
Total number of unique CDR3s TCRA TCRB	3450 3986	2747 1584
Top 10 TRV genes *TRAV* (%) *TRBV* (%)	46.04 45.56	42.61 40.28

The number of detected unique clone counts per sample ranged from 0 to 191 for TCRA and 0 to 190 for TCRB by TRUST4 (**Figures 1E, F**), and 0 and 134 TCRA and 0 and 69 TCRB by MIXCR (**Figures 1G, H**) and most samples (>85%) had ≤50 identified clones. The average number of unique clones per patient was 24.8 ± 28.6 and 27.4 ± 33.4 for TCRA and TCRB, respectively (TRUST4), and 18.9 ± 21.6 and 10.8 ± 13.2 for TCRA and TCRB, respectively, (MIXCR). We identified a total of 3450 unique TCRA CDR3s and 3986 unique TCRB CDR3s by TRUST4 (**Table 1**), while 2747 unique TCRA CDR3s and 1584 unique TCRB CDR3s were identified by MIXCR (**Table 1**). Only 1389 TCRA (28.9% of total number of CDR3s) and 1327 TCRB CDR3s (31.3% of total number of CDR3s) were shared by the two computational tools (**Figures 1I, J**).

The CDR3 length of TCRA was shorter in clones extracted using TRUST4 than clones extracted with MIXCR (38.9 ± 6.1 nt or 12.9 ± 2.0 aa vs 40.3 ± 5.2 nt or 13.5 ± 1.8 aa). While the opposite was observed for TCRB (41.6 ± 5.6 nt or 13.9 ± 1.9 aa from TRUST4 vs. 40.6 ± 4.6 or 13.5 ± 1.5 aa for MIXCR) (**Figures 1K, L**).

We found a strong correlation of the frequencies of the shared CDR3s between TRUST4 and MIXCR for TCRA (r=0.513, p<0.001, **Figure 1M**) and TCRB (r=0.345, p<0.001, **Figure 1N**). In addition, both TRUST4 and MIXCR data showed a strong correlation between the numbers of unique clones for TCRA and TCRB per patient (r=0.944, p <0.001 for TRUST4, r=0.941, p <0.001 for MIXCR, **Figures 1O, P**).

### Identification of public CDR3 clones

**Figure 2:**
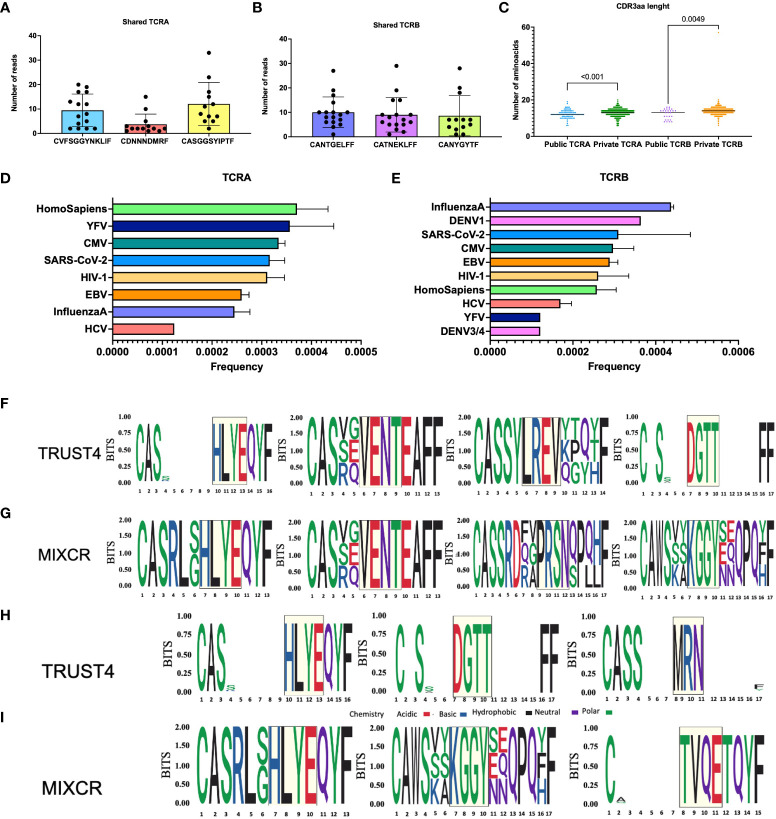
Analysis of common motifs and TCRs with known antigen specificities. Top 3 public TCRA **(A)** and TCRB **(B)**, each dot represents one patient, CDR3aa length comparison between public and private TCR clones **(C)**. Frequencies of TCRB with known antigen specificities **(D)**. Frequencies of TCRB with known antigen specificities **(E)**. TRUST4 TCRB motifs identified by GLIPH2 based on CD4^+^ reference **(F)**. MIXCR TCRB motifs identified by GLIPH2 based on CD4^+^ reference **(G)**. TRUST4 TCRB motifs identified by GLIPH2 based on CD8^+^ reference **(H)**. MIXCR TCRB motifs identified by GLIPH2 based on CD8^+^ reference **(I)**. The relative size of each aa symbol is proportional to its frequency in the dataset, while the total height of aa symbols indicates the probability of the specific aa in the position in bits. White gaps present in the logos represent common deletions and/or insertions in the position.

We searched the complete TCR repertoire in the whole cohort to identify the CDR3 clones from TRUST4 that were shared by more than one individual, called public CDR3, based on sharing identical nucleotide and amino acid sequences. We found that the top 3 public TCRA clones have the following CDR3 amino acid sequences CVFSGGYNKLIF, CDNNNDMRF, and CASGGSYIPTF (**Figure 2A**). For TCRB CDR3, the top 3 public CDR3 clones were CANTGELFF, CATNEKLFF, and CANYGYTF (**Figure 2B**). The CDR3 length was found to be shorter for the public CDR3 clones compared with the private CDR3 clones (TCRA: 12 vs 13, p<0.001; TCRB: 13 vs 14, p=0.005, **Figure 2C**).

Further we aligned TCR sequences against VDJdb records to assess TCR antigen specific CDR3s in patients with AML. We found epitopes belonging to YFV, CMV, SARS-CoV-2, HIV-1, EBV, Influenza and HCV shared between TCRA and TCRB repertoires and less common DENV3/4 and DENV1 epitopes present in TCRB repertoire (**Figures 2D, E**).

We clustered the TCRB clonotypes with high probability of having shared antigen specificities in TCRB repertoires from all patients with AML using GLIPH2. We found HLYE and VENT motifs to be shared by MIXCR and TRUST4 data (**Figures 2F, G**), while LREV and DGTT were uniquely identified from TRUST4, and PRSN and KGGY were only identified from MIXCR when CD4^+^ reference was used. When CD8^+^ was used as a reference we identified HLYE motifs shared between the two tools while DGTT and MRN were additionally identified from TRUST4 and KGGY and TVQE motifs were identified from MIXCR data (**Figures 2H, I**). Further search for TCR CDR3s containing these common motifs using VDJdb revealed that TVQE and PRSN are present in CDR3s associated with Influenza A, HLYE with HCV, while others are all found in CDR3s associated with CMV among other viral epitopes.

### The number of unique TCR clones is correlated with patients’ clinical characteristics

The absolute number of unique clones is not a good TCR measure since it is influenced by the sequencing depth and T cell content across samples. Therefore, we mainly focused on the normalized number of unique clones, in which the number of unique clones was divided by sequencing reads and 1-PB, except in analyses where PB was included in as a variable, the numbers of unique clones were normalized by sequencing reads only.

To assess whether features of the TCR repertoire are associated with a patients' clinical characteristics, we compared normalized number of unique clones for TCRA and TCRB according to each clinical parameter (**Table S1**).

**Figure 3:**
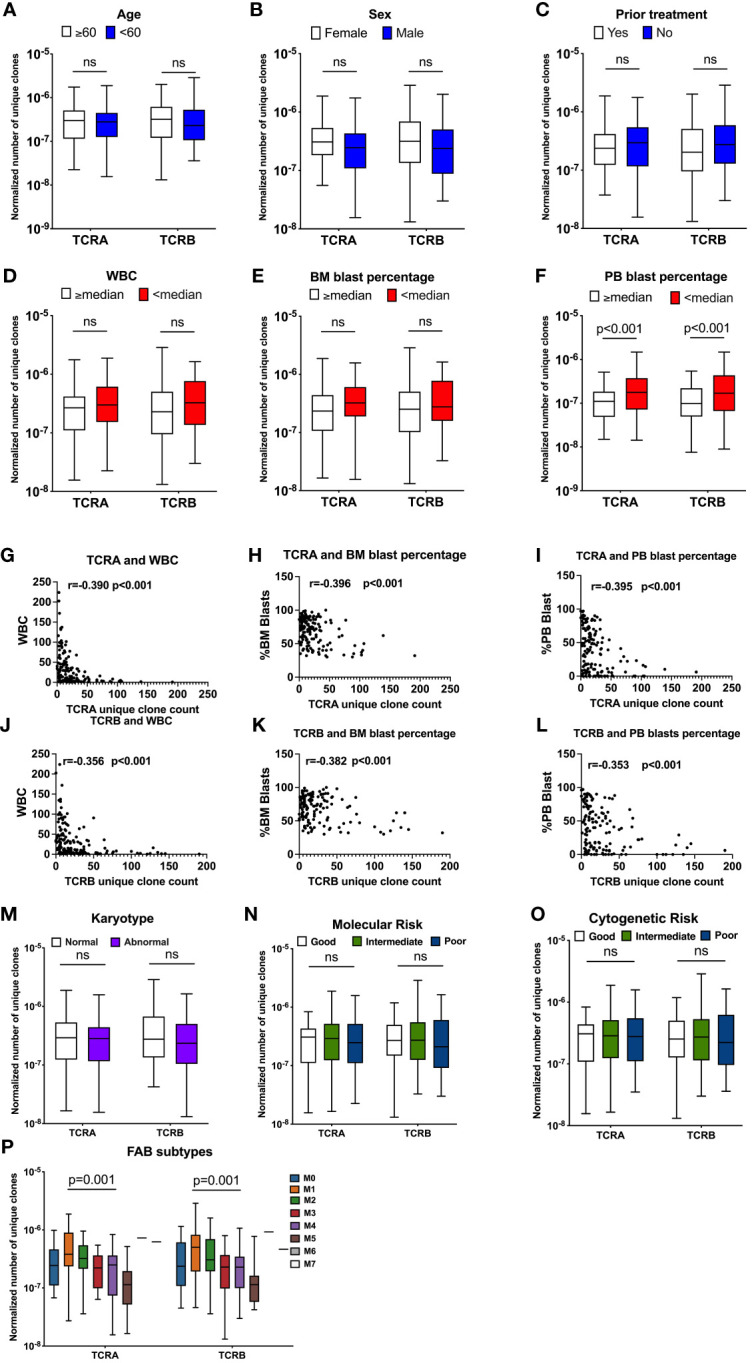
TCR unique clones are correlated with patients’ clinical characteristics. Association between normalized number of unique TCR clones and age (**A**, n=141), sex (**B**, n=141), prior treatment (**C**, n=141), WBC (**D**, n=141), BM blast percentage at diagnosis (**E**, n=141), Percentage of PB blasts at diagnosis (**F**, n=141, TCRA: q=0.002, TCRB: q=0.004). Pearson correlation between unique TCRA clone count and WBC **(G)**, Percentage of BM blasts at diagnosis **(H)** Percentage of PB blasts at diagnosis **(I)**, TCRB and WBC **(J)** Percentage of BM blasts at diagnosis **(K)**, Percentage of PB blasts at diagnosis **(L)**. Association between normalized number of unique clones and: Karyotype (**M**, n=138), Molecular Risk (**N**, n=138), Cytogenetic Risk (**O**, n=138), FAB subtypes (**P**, n=140). Data were analyzed by unpaired t test on log(Y) transformed data or Kruskal-Wallis test with Dunn’s posy-hoc test with p<0.05 showing significant difference between groups. FDR correction for multiple comparisons by the Benjamini-Hochberg procedure was performed and considered significant when q value is <0.05, ns, not significant.

The normalized number of unique clones was not significantly different between younger and older patients (**Figure 3A** and **Figure S2A**). We observed that female patients had a trend for a higher, yet not statistically significant normalized number of unique TCR compared with male patients (normalized number of unique clones TRUST4: TCRA: 3.08x10^-7^ vs 2.46x10^-7^, p=0.02, q=0.08; TCRB: 3.16x10^-7^ vs 2.39x10^-7^, p=0.14, q=0.31, **Figure 3B**; MIXCR: TCRA 2.30x10^-7^ vs 2.09x10^-7^, p=0.11, q=0.26; TCRB: 1.31x10^-7^ vs 9.50 x10^-8^, p=0.05, q=0.16 **Figure S2B**). We observed no difference in the normalized number of unique TCR clones between patients according to whether they received prior treatment or not (**Figure 3C** and **Figure S2C**).

When patients were dichotomized by median WBC, BM or PB blast percentage, we found a trend towards a lower normalized number of unique TCR clones in patients with WBC above or equal to the median (TRUST4: TCRA: 2.68x10^-7^ vs 2.98x10^-7^, p=0.06, TCRB: 2.29x10^-7^ vs 3.24x10^-7^, p=0.08, **Figure 3D**, MIXCR: TCRA: 2.04x10^-7^ vs 2.40x10^-7^, p=0.07; TCRB: 1.02x10^-7^ vs 1.14 x10^-7^, p=0.28, **Figure S2D**) compared with patients with WBC lower than the median. We observed a suggestive lower normalized number of unique clones for TCRA (TRUST4: TCRA: 2.34x10^-7^ vs 3.23x10^-7^, p=0.06 **Figure 3E**, MIXCR: TCRA: 1.70x10^-7^ vs 2.40x10^-7^, p=0.24, **Figure S2E**) but not TCRB (**Figure S3E** and **S2E**) in patients with above or equal to the median BM blast percentage compared with patients that had lower than median BM blast percentage at diagnosis, however those values did not meet FDR error correction. A significantly lower normalized number of unique TCR clones was observed in patients that had higher or equal to the median PB blast percentage compared with patients that had lower than median PB blast percentage (TRUST4: TCRA: 1.11x10^-7^ vs 1.77x10^-7^, p<0.001; q=0.002 TCRB: 9.84x10^-8^ vs 1.70x10^-7^, p<0.001, q=0.004 **Figure 3F**, MIXCR: TCRA: 7.93x10^-8^ vs 1.33x10^-7^, p<0.001, q=0.002; TCRB: 4.09x10^-8^ vs 7.06x10^-8^, p<0.001, q=0.002 **Figure S2F**).

To better understand this observed association between the TCR repertoire with the disease specific characteristics, we assessed the correlation between the number of unique TCR clones with disease specific patients' characteristics including WBC, BM blast percentage, and PB blast percentage. As expected, we found a strong negative correlation between the unique TCR clones and WBC (p<0.001), BM blasts percentage (p<0.001), and PB blast percentage (p<0.001) (**Figures 3G-L** and **S2G-L**).

No association was found between TCR features according to karyotype, molecular risk, or cytogenetic risk. These data were summarized in **Table S1** and **Figures 3M-O** and **S2M-O**. Normalized number of unique TCRA and TCRB clone counts were significantly different among French-American-British (FAB) classes (TRUST4: TCRA: p<0.001, TCRB: p=0.001, **Figure 3P**; MIXCR: TCRA: p<0.001, TCRB: p<0.001, **Figure S2P**), with the lowest normalized unique TCR clones observed for the M5 and the highest for the M1.

Consistently univariate regressions of TRUST4 normalized to read only number of unique TCR clones with clinical characteristics of AML patients are summarized in **Table 2** and showed that sex, prior treatment, WBC, BM blast percentage and PB blast percentage were individually associated with the normalized to reads only number of TCR unique clones.

**Table 2:** Univariate regression of TRUST4 normalized number of unique TCR clones with clinical characteristics of AML patients.

Characteristics	TCRA clone median (x10^-7^)	p	TCRB clone median (x10^-7^)	p
**Age** Young (<60) Old (≥60)	1.14, n=78 1.47, n=65	0.217	1.14, n=78 1.66, n=64	0.076
**Sex** Male Female	1.45, n=78 1.56, n=65	0.017	1.00, n=78 1.38, n=64	0.055
**Prior treatment** Yes No	0.81, n=34 1.46, n=109	0.008	0.82, n=33 1.56, n=109	0.005
**WBC count ** Above median Below median	0.74, n=72 2.21, n=71	<0.0001	0.70, n=71 2.21, n=71	<0.0001
**BM blast percentage** Above median Below median	1.04, n=73 1.55, n=70	0.005	1.14, n=74 1.61, n=68	0.002
**PB blast percentage** Above median Below median	1.07, n=72 1.96, n=69	<0.0001	0.98, n=72 1.70, n=69	0.0005
**Karyotype** Normal Abnormal	1.45, n=59 1.23, n=81	0.981	1.47, n=59 1.14, n=80	0.803
**Risk (Molecular)** Good Intermediate Poor	1.30, n=30 1.48, n=72 1.13, n=38	0.644	1.60, n=29 1.25, n=72 0.83, n=38	0.266
**Risk (Cytogenetic)** Good Intermediate Poor	1.25, n=29 1.43, n=76 1.16, n=35	0.960	1.60, n=28 1.28, n=76 0.83, n=35	0.467
**FAB** M0 M1 M2 M3 M4 M5 M6 M7	1.02, n=14 1.45, n=35 1.55, n=34 1.77, n=15 1.19, n=27 1.01, n=15 7.27, n=1 5.87, n=1	0.911	0.86, n=14 1.27, n=36 1.60, n=33 2.02, n=14 1.25, n=27 0.82, n=15 9.26, n=1 4.36, n=1	0.906

p values were assigned on log(Y) transformed data. Data were normalized to reads only.

### Association of the numbers of unique TCR clones with AML mutations

**Figure 4:**
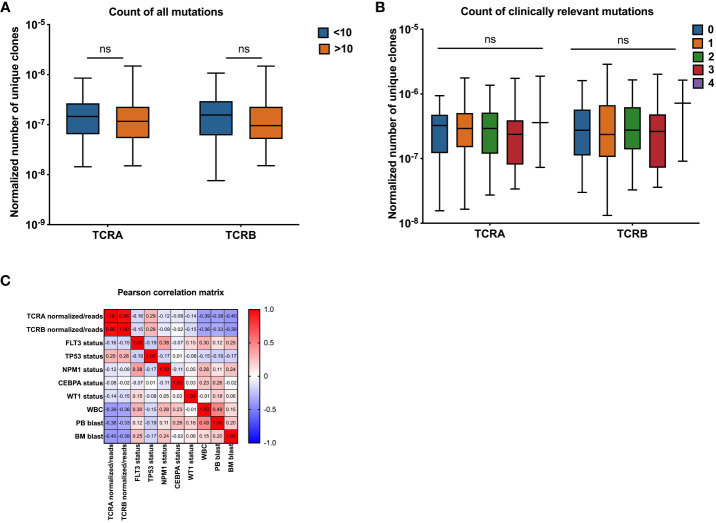
Association between normalized number of unique TCRA and TCRB clones and common AML mutations. Association between normalized number of unique clones with count of all mutations (**A**, n=141), clinically relevant AML mutations (**B**, n=141), Pearson correlation between number of unique TCR clones normalized to the total number of sequencing reads and patients clinical and molecular characteristics **(C)** Data were analyzed by Kruskal Wallis test with Dunn’s *post-hoc* test with p<0.05 showing significant difference between groups, ns, not significant.

We assessed the association between TCR features and patients’ mutational burden. We did not find differences between patients with highly mutated disease compared with patients that have low number of mutations (**Figures 4A** and **S3A**). No differences were observed when only the most common mutations in AML (*DNMT3*, *FLT3*, *NPM1*, *TET2*, *RUNX1*, *IDH1/2*, *TP53*, *CEBPA*, *NRAS*, *WT1*) were considered. Patients were grouped according to the number of different mutations they had into 0, 1, 2, 3 and 4, we did not find significant differences between groups (**Figures 4B** and **S3B**).

We also assessed the TCR features according to the mutational status of each common mutation in AML. Among the most common AML mutations we found that *FLT3* and *IDH1* mutations had a suggestive, yet not statistically significant association with the normalized number of unique clones when comparing medians between patients carrying wild type gene versus the mutant (**Table S2**, **Figures S4J, K** and **S5J, K**).

Univariate regression of TCRA and TCRB median normalized number of unique clones with mutational status of AML patients using TRUST4 data showed significant differences in T-cell receptor clones between mutant and wild-type groups for some genes in AML patients. Specifically, significant differences were observed for both TCRA and TCRB median values in *FLT3* and *TP53* mutant groups, for TCRA median values in *NPM1* and *CEBPA* mutant groups and for TCRB median values in *WT1*. However, no significant differences were observed for other mutations such as *DNMT3A*, *TET2*, *RUNX1*, *IDH2*, *IDH1*, and *NRAS* (**Table 3**).

**Table 3:** Univariate regression of TCRA and TCRB median normalized number of unique clones with mutational status of AML patients using TRUST4.

GENE	TCRA median (x10^-7^)	P value	TCRB median (x10^-7^)	P value
*DNMT3A* Mutant WT	1.04, n=35 1.34, n=108	0.495	1.07, n=34 1.25, n=108	0.381
*FLT3* Mutant WT	0.70, n=43 1.55, n=100	0.004	0.73, n=42 1.29, n=100	0.020
*NPM1* Mutant WT	0.77, n=35 1.43, n=108	0.044	1.07, n=36 1.26, n=106	0.087
*TET2* Mutant WT	1.89, n=12 1.25, n=131	0.940	1.44, n=12 1.22, n=130	0.804
*RUNX1* Mutant WT	1.53, n=14 1.31, n=129	0.259	1.19, n=14 1.23, n=128	0.485
*IDH2* Mutant WT	1.49, n=16 1.20, n=127	0.942	1.43, n=16 1.22, n=126	0.749
*TP53* Mutant WT	3.23, n=11 1.24, n=132	0.010	3.05, n=11 1.14, n=131	0.011
*CEBPA* Mutant WT	0.35, n=12 1.34, n=131	0.018	0.46, n=11 1.24, n=131	0.073
*IDH1* Mutant WT	1.56, n=13 1.25, n=130	0.327	1.39, n=14 1.22, n=128	0.253
*NRAS* Mutant WT	0.47, n=5 1.33, n=138	0.828	0.52, n=6 1.24, n=136	0.843
*WT1* Mutant WT	0.72, n=9 1.35, n=134	0.080	0.57, n=9 1.24, n=133	0.043

p values were assigned on log(Y) transformed data.

The univariate and multivariable regressions including both clinical and molecular parameters were performed. We introduced the model that included all significant features with TCRA and TCRB as separate outcomes (**Table 4**). PB blast percentage was eliminated by the model selection procedure, due to a strong correlation with WBC and BM blast percentage (**Figure 4**). Interestingly we found *WT1*, WBC and BM blast percentage to be negatively associated with TCRA and TCRB, while the associations that we found in the univariate analysis were lost for *FLT3, NPM1* and *TP53* which is likely due to their strong correlation with WBC and BM blast percentage. Pearson correlation heatmap is presented in **Figure 4C** and corresponding p values in **Table S3**.

**Table 4:** Multivariable regressions of TCRA and TCRB median normalized number of unique clones with mutational status and patient characteristics of AML patients using TRUST4.

Characteristics	TCRA median (x10^-7^) Log-Transformed				TCRB median (x10^-7^) Log-Transformed			
	Parameter Estimate ± SE	P value for each factor	F test	P value of F test	Parameter Estimate ± SE	P value for each factor	F test	P value of F test
WT1 Sex WBC count BM blast percentage	-0.589 ± 0.296 0.371 ± 0.144 -0.012 ± 0.002 -0.013 ± 0.004	0.04 0.01 <0.0001 0.0009	19.05	<0.0001	-0.697 ± 0.322 0.264 ± 0.158 -0.011 ± 0.002 -0.014 ± 0.004	0.03 0.09 <0.0001 0.001	13.73	<0.0001

### The numbers of unique TCR clones are highly correlated with the expression of T cell exhaustion markers

**Figure 5:**
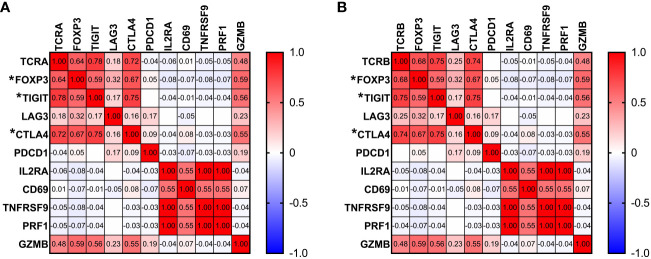
Associations between TCR repertoire and T cell markers. Pearson correlation between gene expression of T cell markers and number of unique TCRA **(A)** and TCRB **(B)** clones. Correlation with p<0.0001 is marked by “*” next to the gene name.

To better understand how the state of T cells in the context of AML relates to the TCR repertoire, we analyzed associations between unique number of TCRA or TCRB clonotypes and gene expression of T cell markers. No correlation was found between activation markers such as *IL2RA*, *CD69* or *TNFRSF9* and TCR repertoire (**Figures 5A, B**). We observed a strong positive correlation between T regulatory cell marker *foxp3* and both TCRA and TCRB (TRUST4, TCRA r=0.64, p<0.0001; TCRB r=0.68, p<0.0001, **Figures 5A, B**). Furthermore, T cell inhibitory signal genes such as *TIGIT*, *LAG3* and *CTLA4* also show positive correlation with the number of unique TCRA (*TIGIT*: r=0.78, p<0.0001; *LAG3*: r=0.18, p=0.029; *CTLA*: r=0.72, p<0.0001, **Figure 5A**) and TCRB clones (*TIGIT*: r=0.75, p<0.0001; *LAG3*: r=0.25, p=0.003; *CTLA*4: r=0.74, p<0.0001; **Figure 5B**). No correlation was identified for TCRA or TCRB with *PDCD1 or PRF1*. However, expression of *GZMB* was also strongly correlated with the number of unique TCRA and TCRB (TCRA: r= 0.48, p<0.0001; TCRB: r=0.48, p <0.0001, **Figures 5A, B**).

### Association between public clones and overall survival

To determine whether TCR unique clones are associated with the patients' clinical outcome, overall survivals were compared between patients that have higher and lower than median normalized number of unique TCRA or TCRB. No significant difference was observed for patients with higher than median compared with patients with lower than median unique TCRA or TCRB clone count or when comparing patients from each quartile of unique TCRA or TCRB clone counts. Also, no differences were observed when normalized unique TCRA or TCRB clone count was used as a continuous variable in the survival analysis.

We also examined whether a particular top 3 shared CDR3 sequence is associated with overall survival. We found that patients that carried the CANTGELFF TCRB CDR3 clone (N=16) had 55.4 months overall survival (OS) compared with 19 months for patients that did not have it (N=129) (p=0.05, q=0.15) suggestive of a better OS. No other top 3 shared CDR3s were associated with overall survival. However public clones detected by TRUST4 were not consistent with MIXCR suggestive of possible false-positive findings.

## Discussion

Characteristics of the TCR repertoire such as diversity indices or clonal expansion and/or composition reflect the antigen specificity and T cell dynamic changes. Highly expanded clones within tumors may contribute significantly to the antitumor response as previously observed in different types of cancer (29). Several tools that leverage the RNA-seq data to extract TCR repertoire data have been developed (22, 23). RNA-seq data are capable of profiling dominant clonotypes with high frequencies but will unlikely capture the diversity of the repertoire or identify the less abundant clones. Li et al. developed TRUST (19), which then was employed by the group on both pediatric and adult AML samples to characterize T and B cell receptors (TCR and BCR) repertoires (10). The study showed lower number of normalized TCR CDR3 counts in infants (0-3 years old) and children (3-20 years old) when compared with healthy children, and in adults (over 20 years old) with AML when compared with healthy adults. Although the study has explored the association between TCR repertoire characteristics and major common AML mutations and clinical outcome, a comprehensive analysis of how TCR repertoire features correlate with both clinical and molecular characteristics of patients with AML remains to be fully elucidated. Here we applied an updated version of TRUST (TRUST4), previously found to have significantly improved computational efficiency and the number of recovered CDR3s (30) along with another recently developed tool (MIXCR) to compare their output and comprehensively examine the TCR repertoires in the adult AML cohort and how their features are associated with patients’ molecular and clinical characteristics based on available RNA-seq database.

To ensure robustness of the analysis we applied two of the most commonly used computational tools to extract TCR information from RNA-seq data – TRUST4 and MIXCR. These tools were previously benchmarked with other computational tools to extract TCR information from RNA-seq data originating from the same samples focusing on the characterization of T-cell rich and T cell poor tissues. Both methods were found effective in capturing estimated repertoire diversity in T-cell rich tissues particularly in samples with a limited number of hyperexpanded clones (31). However, the exact accuracy of the alignment from TRUST4 and MIXCR remains unknown due to the lack of a control framework of known TCR composition both quantitatively and qualitatively for direct comparison and assessment. This highlights the need to establish rigorous controls that can allow for identification of potential disparities in alignment accuracy to allow for instilling confidence in the reliability of T-cell receptor analysis output. The average numbers of TCRA and TCRB reads from RNA-seq were higher in TRUST4 output compared with MIXCR output. Both tools have resulted in satisfactory coverage of *TRAV* and *TRBV* genes capturing 65-75.5% of the V and 76-86% of the J known genes. While TRUST4 has a similar number of TCRA and TCRB reads on average, MIXCR has a higher number of reads for TCRA than TCRB and less coverage across *TRBV* segment usage. Mapped reads obtained from TRUST4 and MIXCR were used to estimate the frequency of different *TRAV* and *TRBV* genes across a cohort of patients with AML. Among the top 10 V genes identified in a previous study on 29 cancer types from the TCGA data (19), TRUST4 identified six *TRAV* and six *TRBV* and MIXCR identified five of *TRAV* and seven of *TRBV* genes (19) in the AML cohort. Small overlap between clonotypes identified by TRUST4 and MIXCR suggest differences in the performance of the two computational tools and highlights that study design needs to be carefully considered when applying these tools on RNA-seq data. The alignment algorithm is significantly different between TRUST4 and MIXCR and detailed explanation of each alignment method is reported in detail by their developers (17, 22, 23). Differences in V gene assignment algorithms and CDR3 amino acid sequence start points can lead to discrepancies in identified CDR3 sequences. Additionally, variations in alignment methods and contig generation can impact the number of aligned reads per patient. To ensure confidence in the associations between TCR features and molecular/clinical characteristics, we utilized the two computational tools. While both tools showed similar patterns, only approximately 30% of the identified CDR3 sequences were shared between them. This discrepancy makes interpreting data containing specific sequences and quantitative information of certain clones challenging, as the tools assemble different clonotypes.

We observed a slightly higher normalized number of unique clones in female patients compared with males, an observation that was seen in prior studies (19, 32). We also found that clinically relevant characteristics such as WBC, BM blast percentage and PB blast percentage negatively correlate with TCRA and TCRB unique clone counts and therefore patients with higher than median white blood cell count, percentage of bone marrow blasts and percentage of PB blasts have been found to have lower normalized number of unique clones. One possible explanation for this association is that leukemic blasts may outcompete normal T cells, leading to a reduction in the number of unique T cell clones in the sample. Similarly, a higher WBC count may indicate a more aggressive disease with a higher burden of leukemic blasts, which in turn may result in a lower number of T cells and therefore fewer unique T cell clones. Skewed TCR repertoire may be related to a response to myeloblast microenvironment, however it was previously suggested that while T cells may recognize cancer cells, effective antitumor response is impaired.

Previous studies found a strong positive association between the normalized number of unique clones and the number of nonsynonymous mutations in 29 cancer types from TCGA including cancers with high mutational burden (19). This is possible due to higher number of neoantigens being presented for the T cells (19). We did not observe such association when the number of the most common AML mutations and unique TCRA or TCRB clones were considered. Previous study using TCGA dataset investigated five genes of high clinical significance (*FLT3*, *NPM1*, *KIT*, *CEBPA* and *WT1*), along with three gene fusions (*RUNX1-RUNX1T1*, *CBFB-MYH11*, and *PML-RARA*). Only *CBFB-MYH11* was found to be associated with significantly lower TCRB CPK value in pediatric patients with AML, similar but not significant trend was found in infant and adult patients with AML (10). We found that certain mutations, such as *FLT3* and *NPM1* were associated with a decreased normalized number of unique T cell clones while *TP53* was associated with increased normalized number of unique TCR clones. However, these associations were lost when considering the WBC, BM blasts and PB blasts. This is likely due to the fact that these mutations are known to be associated with these clinical features (33–38). Mutations in *WT1* however were associated with the TCR repertoire when adjusting by other clinical characteristics. It was previously shown that WT1-specific T cells can be generated to target leukemia cells with limited effect on normal progenitor cells (39). Similarly, WT1-specific CD8 T cells were found to be induced in azacitadine and donor lymphocyte infusion treatment contributing to graft versus leukemia effect in MDS and AML patients (40). The presence of WT1-specific CD8 T cells was previously suggested to contribute to maintenance of complete remission (41). Vaccination with peptides derived from WT-1 induce immune responses resulting in an improved clinical outcome (42).

Specific features of the TCR repertoire such as the TCR diversity and clonal expansion were found to correlate with patients’ clinical characteristics, disease status and clinical outcome in various cancers (10, 43–45). Diversity of the TCR repertoire was found to be inversely correlated with age in patients with breast cancer (46). Lower diversity indices were observed in advanced disease stages (46) and upon anti-CTLA-4 therapy in melanoma patients (47). Higher usage of *TRBV20.1* was reported in the HER2- patients during complete pathological remission while *TRBV30* was higher in HER2+ patients responding to trastuzumab (46). Tumor-infiltrating T cell repertoire was more diverse compared with normal pancreatic tissue in pancreatic ductal adenocarcinoma suggesting recognition of tumor derived antigens (48). Higher TCR diversity was found to be correlated with better progression free interval in a few cancer types (49).

Our analysis found no statistically significant difference in overall survival between patients with a lower versus higher than median normalized number of unique clones for both TCRA and TCRB. However, we observed that patients that had CANTGELFF within the identified TCRB clones by TRUST4 tended to have better but not statistically significant overall survival compared with those without such CDR3. According to the TCRdb database the CANTGELFF was previously found in patients with breast cancer, urothelial cancer, healthy subjects and in patients with CMV infection among others (50). However, this finding was not corroborated by the results obtained from MIXCR. Additionally, MIXCR failed to detect public clonotypes shared by more than 3 patients. Consequently, we believe that while both MIXCR and TRUST4 are suitable for analyzing associations between TCR diversity estimates and molecular/clinical patient characteristics, they may not be the most appropriate tools when the analysis involves the identification and quantification of specific CDR3 sequences. To validate our findings, confirmation through TCR targeted sequencing in an independent AML dataset and functional studies are necessary. The observed discrepancy underscores the need for appropriate benchmarking of current algorithms against a known ground truth - a library of TCR receptors with known composition. Regrettably, such a control library is not yet available, hindering the use of RNA-seq data for both quantitative and qualitative analyses in this context.

While the focus of our study was on the association between TCR repertoire and clinical and molecular features in AML patients, the identification of viral epitopes in our analysis is an interesting finding that may have implications for the understanding of the role of viral epitopes in AML patients. Association of CMV infection with AML relapse after allogeneic hematopoietic stem cell transplant (HCT) has been previously identified along with an increase of multifunctional CMV-specific T cells (51, 52). Also, CMV reactivation is a frequent complication after HCT. Even though previous studies have not found association of EBV with the pathogenesis of AML, it has been suggested that it is a secondary event related to compromised immune systems in AML (53). No significant associations were found for HCV, however, it was found to be increased in 8% of patients with AML (54). It is important to note that our epitope analysis approach has some limitations, including the potential for false positives and the inability to distinguish between TCR clones that are specific to the viral epitope versus those that are cross-reactive with other antigens. For example, we identified SARS-CoV-2 related epitopes even though the patient samples are pre-pandemic, however, it is now known that cross-reactive epitopes were present in individuals before the SARS-CoV-2 pandemic (55). It was also previously reported that T cells could cross-react with both CMV and SARS-CoV-2 in SARS-CoV-2 unexposed individuals. Functional studies have shown non-naive spike and non-spike specific T cells in the blood of pre-pandemic individuals with several HLA backgrounds (56–58). Interpretation of antigen-specificity predictions indicating virus-related targets is based on clustering analysis to identify similar specific TCR sequence in a reference dataset. While this database lacks AML-specific TCRs, RNA-seq is only able to capture the most abundant clones due to its shallow nature indicating that these clones were expanded within these patients; therefore it would be suggested that virus-specific memory T cells were leveraged for anti-AML response through T-cell cross-reactivity. Virus specific T cells were previously shown to populate repertoire of human healthy tissues and tumors where they can be leveraged to trigger cytotoxicity in the tumor (59, 60). To confirm whether the virus-specific memory T cells identified in AML patients present an anti-AML response through T-cell cross reactivity versus just background dominance of those TCR clones, functional studies and deep TCR sequencing would be needed. Also, further validation studies would be needed to confirm the clinical relevance of the identified viral epitopes in AML patients.

One limitation of our study is the reliance on bulk RNA-seq data, which unfortunately lacks information on the pairing between TCRA and TCRB. Consequently, this hinders our ability to fully predict the functional receptor responsible for antigen binding. Although single-cell sequencing is the most informative and suitable approach for confidently pairing TCRA and TCRB, previous research has demonstrated that the beta chain of the CDR3 sequence holds the most significant influence on antigen specificity (61).

Public TCRs were previously described in infectious diseases, cancer and autoimmune disease and were found to associate with clinical outcome (62–67). Consistently with previous studies we found that public CDR3s have shorter amino acid length (68). It was previously demonstrated that shorter CDR3 length is related to smaller number of N-insertions including CDR3s specific to viral epitopes such as CMV and EBV (69). In our study we identified CANTGELFF CDR3 which resulted from the recombination of either *TRBV12-3*, *TRBV2*, *TRBV28*, *TRBV30*, *TRBV3-1*, *TRBV5-5*, *TRBV5-7*, *TRBV6-6*, *TRBV7-2* or *TRBV9* with *TRBJ2-2* in patients with a better overall survival. However, this finding was not reproduced using MIXCR and can possibly be a false-positive. Further analysis recognizing public TCRs in patients with AML might be beneficial to recognize leukemia specific TCRs.

AML is characterized by increased prevalence of immunosuppressive phenotype leading to impaired immune response (70–74). Single cell RNA-seq analysis suggested altered T regulatory cells as feature of newly diagnosed AML patients (11). In agreement with the previous studies we observed strong positive correlation between *foxp3* expression and the number of unique TCRA and TCRB. Studies on AML animal models suggest impaired T cell function with features of exhaustion or antigen-specific T cell tolerance (75–77). Anergy is then developed through lack of co-stimulatory signals with excessive stimulation of the TCR and high levels of inhibitory signals such as *PD-1, CTLA4, TIGIT, LAG3* (78). Consistently we found that inhibitory signals such as *TIGIT* and *CTLA-4* were found to be positively correlated with unique number of TCR clones. Interestingly *PDCD1* was found not to be associated with TCR repertoire. Further high positive correlation of *GZMB*, but not *PRF1* with the number of unique TCRA and TCRB was observed suggesting association between cytotoxic T cells and TCR repertoire. Observed correlations suggest more exhausted T cell profile in characterized patients with AML consistently with previous research (11). Although exhausted CD8 T cells can still identify antigens through TCR, they do not effectively respond to the antigen (79). Lack of correlation with perforin and correlations with co-inhibitory receptors but not activation markers further supports dysfunctional immune microenvironment within patients with AML.

Several limitations of this study are present. AML is a heterogeneous hematological malignancy; the relatively small sample size might not fully reflect all aspects of this disease. Secondly, RNA-seq data provides a shallow estimation of the TCR repertoire, as very limited number of clones and low number of reads corresponding to the most abundant clones, and the broader TCR repertoire cannot be captured. Low number of reads does not allow for calculation of features of TCR repertoire such as diversity indices or detection of rare clonotypes therefore we used normalized unique clone count as an approximate metric for TCR diversity. While we analyzed public clonotypes there is a chance we missed some individuals also sharing the same clonotypes shared in this population due to low coverage. Associations made between TCR repertoire features and clinical and molecular characteristics are mainly based on abundant clones which are more expanded in comparison to not detected rare clonotypes; therefore full representation of the repertoire is not accessible. If certain antigen specific clonotypes related to certain mutations are less abundant they might not be detected here which would affect the mutational data analysis. Furthermore 447 out of 2820 TCRA clones and 830 out of 1603 TCRB clones MIXCR were assigned to multiple *TRAV* or *TRBV* genes possibly due to the short read length. The small percentage of overlap between clones identified by TRUST4 and MIXCR further highlights the need for benchmarking studies that validate these computational methods. Furthermore, although we identified top 10 *TRAV* and *TRBV* exons, only single cell sequencing would be able to determine which clones are paired to form a functional TCR receptor consisting of alpha and beta chains.

Despite the limitations of low coverage associated with the use of RNA-seq data to extract TCR sequence information and the small sample size, we were able to report statistically significant associations between the TCR repertoire and clinical and molecular features in patients with AML. Our findings highlight the importance of analyzing the TCR repertoire in AML to better understand the adaptive immune response against leukemic cells and the impact of the disease on the immune repertoire and the later on the clinical outcome.

Our study revealed unexpectedly poor reproducibility of detected CDR3 clones between the two software packages. This finding is important because it highlights the need for caution when interpreting results obtained using different software tools such as those focusing on the specific expanded clones or the V segment utilizations. Nevertheless, for the type of correlative analyses we performed, the two packages performed very similarly and showed the same patterns of associations observed between the TCR repertoire and clinical and molecular features were consistent. This consistency supports the robustness of our findings and suggests that the associations we reported are likely to be biologically relevant.

While the utilization of RNA-seq data enables the identification of highly abundant clonotypes in patient samples, it is important to acknowledge the limitations associated with the low coverage of this approach. The study’s findings further highlight the need for TCR-Seq analyses to provide the depth needed to characterize the TCR repertoire in patients with AML accurately. Further, the poor reproducibility in terms of identified CDR3 clones between MIXCR and TRUST4 highlight the need of development of a control of known composition to serve as a biological calibrator for accurate qualitative and quantitative TCR analyses. Future studies using TCR-seq analyses could provide a more comprehensive understanding of the immune response to AML and the potential for TCR-based therapies to improve patient outcomes.

## Data availability statement

Publicly available datasets were analyzed in this study. This data can be found here: https://portal.gdc.cancer.gov/projects/TCGA-LAML.

## Ethics statement

Ethical approval was not required for the studies involving humans because this study was conducted on publicly available dataset. The studies were conducted in accordance with the local legislation and institutional requirements. Written informed consent to participate in this study was not required from the participants or the participants’ legal guardians/next of kin in accordance with the national legislation and the institutional requirements.

## Author contributions

MP: data analysis, validation and visualization, writing-original draft, reviewing-editing, methodology. MT: data curation, software, formal analysis, validation, methodology, writing the original draft. Y-CW: formal analysis, writing-original draft, review, and editing. AK: data curation, software, formal analysis, validation, methodology, writing-review editing. ML and JM: methodology, writing-review editing, and provided statistical input. HA: conceptualization, resources, supervision, funding, validation, writing original draft, project administration, writing-review, and editing. All authors contributed to the article and approved the submitted version.
