# Supplementary material for: Features of the TCR repertoire associate with patients' clinical and molecular characteristics in acute myeloid leukemia
Source: Front Immunol. 2023 Oct 19;14:1236514. doi: 10.3389/fimmu.2023.1236514 (PMC10620936; doi:10.3389/fimmu.2023.1236514)
Supplement: Supplementary file 1 [file DataSheet_1.pdf]

Features of the TCR Repertoire Associate with Patients' Clinical and Molecular Characteristics in Acute Myeloid Leukemia

Authors: Mateusz Pospiech[1], Mukund Tamizharasan[1,4], Yu-Chun Wei[1], Advaith Maya Sanjeev Kumar[1,4], Mimi Lou[1], Joshua Milstein[3], Houda Alachkar[1,2]

Affiliations:

[1]Department of Clinical Pharmacy, School of Pharmacy, University of Southern California, Los Angeles, CA, USA

[2]Norris Comprehensive Cancer Center, University of Southern California, Los Angeles, CA

[3]Department of Population and Public Health Sciences, Keck School of Medicine, University of Southern California, Los Angeles, CA

[4]Department of Computer Science, University of Southern California, Los Angeles, CA

Corresponding Author:

Houda Alachkar, PharmD, Ph.D.

Associate Professor

University of Southern California, School of Pharmacy

1985 Zonal Avenue John Stauffer Pharmaceutical Sciences Center Room 608

Los Angeles CA 90089

Telephone: 323-442-2696

Email: alachkar@usc.edu

Keywords: Acute myeloid leukemia; T cell receptor (TCR); *WT1*; RNA sequencing (RNA-Seq); TCGA

Table of Contents

Supplemental tables: *3*

Table S1 Clinical characteristics of AML patients according to TRUST4 normalized number of unique TCR clones 3

Table S2. Comparison of TCRA and TCRB median normalized number of unique clones and mutational status of AML patients using TRUST4. 4

Table S3. Pearson correlation p values between TCR and clinical and molecular characteristics of patients with AML. 5

Supplemental Figures: *6*

Figure S1 V exon usage heatmaps 6

Figure S2 Normalized number of TCR unique clones is correlated with patients' clinical characteristics. 7

Figure S3. Association between normalized number of unique TCRA and TCRB clones identified by MIXCR and common AML mutations. 8

Figure S4. Association between normalized number of unique clones identified by TRUST4 and common AML mutations. 9

Figure S5. Association between normalized number of unique clones identified by MIXCR and common AML mutations. 10

Supplemental tables:

Table S1 Clinical characteristics of AML patients according to TRUST4 normalized number of unique TCR clones

Characteristics	TCRA clone median ($x10^{-7}$)	p	TCRB clone median ($x10^{-7}$)	p
**Age**		0.658		0.471
Young (<60)	2.78		2.30	
Old (≥60)	2.99		3.20	
**Sex**				
Male	2.46	0.023	2.39	0.138
Female	3.08		3.16	
**Prior treatment**		0.680		0.309
Yes	2.38		2.04	
No	2.95		2.74	
**WBC count**		0.056		0.082
Below median	2.98		3.24	
Above median	2.68		2.29	
**BM blast percentage**		0.064		0.177
Above median	2.34		2.53	
Below median	3.23		2.77	
**PB blast percentage**		<0.001*		<0.001*
Above median	1.11		0.98	
Below median	1.77		1.70	
**Karyotype**		0.461		0.256
Normal	2.92		2.76	
Abnormal	2.84		2.36	
**Risk (Molecular)**		0.771		0.689
Good	3.08		2.70	
Intermediate	2.92		2.72	
Poor	2.48		2.11	
**Risk (Cytogenetic)**		0.907		0.901
Good	3.07		2.53	
Intermediate	2.86		2.72	
Poor	2.78		2.23	
**FAB**		<0.001		0.001
M0	2.44		2.38	
M1	3.81		5.03	
M2	3.23		3.05	
M3	2.22		2.29	
M4	2.49		2.29	
M5	1.14		1.14	
M6	7.27		9.26	
M7	6.25		4.64	

p values were calculated based on log(Y) transformed data by performing unpaired t test.

*meeting FDR error correction performed by the Benjamini-Hochberg procedure.

Table S2. Comparison of TCRA and TCRB median normalized number of unique clones and mutational status of AML patients using TRUST4.

GENE	TCRA median ($x10^{-7}$)	P value	TCRB median ($x10^{-7}$)	P value
DNMT3A		0.512		0.873
Mutant	3.17		2.77	
WT	2.76		2.53	
FLT3		0.023		0.045
Mutant	2.10		2.16	
WT	3.21		2.93	
NPM1		0.180		0.679
Mutant	2.22		2.63	
WT	3.06		2.70	
TET2		0.786		0.891
Mutant	3.08		2.93	
WT	2.86		2.70	
RUNX1		0.610		0.900
Mutant	2.86		2.57	
WT	2.86		2.70	
IDH2		0.565		0.573
Mutant	2.62		2.97	
WT	2.98		2.77	
TP53		0.215		0.200
Mutant	4.35		4.64	
WT	2.85		2.57	
CEBPA		0.644		0.656
Mutant	3.85		3.25	
WT	2.89		2.70	
IDH1		0.022		0.031
Mutant	3.60		5.04	
WT	2.81		2.39	
NRAS		0.448		0.848
Mutant	1.89		2.53	
WT	2.92		2.71	
WT1		0.472		0.335
Mutant	2.34		1.93	
WT	3.02		2.71	

*p values were calculated based on log(Y) transformed data by performing unpaired t test. None of the values were significant when FDR correction for multiple comparison which was performed by the Benjamini-Hochberg procedure

Table S3. Pearson correlation p values between TCR and clinical and molecular characteristics of patients with AML.

	TCRA normalized/reads	TCRB normalized/reads	FLT3 status	TP53 status	NPM1 status	CEBPA status	WT1 status	WBC	PB blast	BM blast
TCRA normalized/reads		<0.001	0.054	0.001	0.174	0.332	0.108	<0.001	<0.001	<0.001
TCRB normalized/reads	<0.001		0.070	0.001	0.289	0.837	0.084	<0.001	<0.001	<0.001
FLT3 status	0.054	0.070		0.024	<0.001	0.384	0.082	<0.001	0.145	0.003
TP53 status	0.001	0.001	0.024		0.048	0.869	0.371	0.069	0.021	0.040
NPM1 status	0.174	0.289	<0.001	0.048		0.211	0.545	0.001	0.189	0.004
CEBPA status	0.332	0.837	0.384	0.869	0.211		0.704	0.006	0.002	0.781
WT1 status	0.108	0.084	0.082	0.371	0.545	0.704		0.864	0.032	0.445
WBC	<0.001	<0.001	<0.001	0.069	0.001	0.006	0.864		<0.001	0.076
PB blast	<0.001	<0.001	0.145	0.021	0.189	0.002	0.032	<0.001		0.015
BM blast	<0.001	<0.001	0.003	0.040	0.004	0.781	0.445	0.076	0.015	

Supplemental Figures:

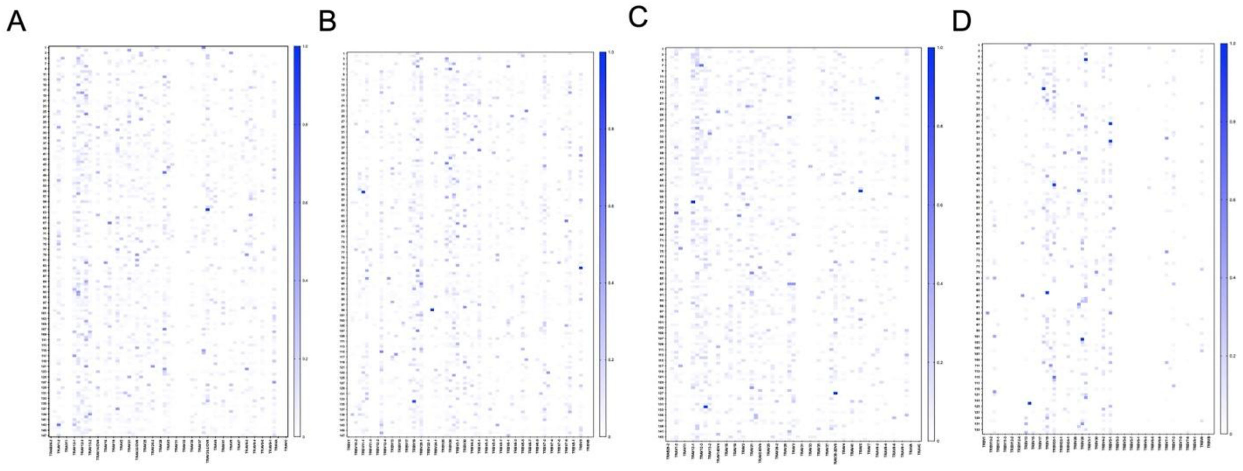

Figure S1 V exon usage heatmaps

A. *TRAV* TRUST 4 B. *TRBV* TRUST4 C. *TRAV* MIXCR D. *TRBV* MIXCR

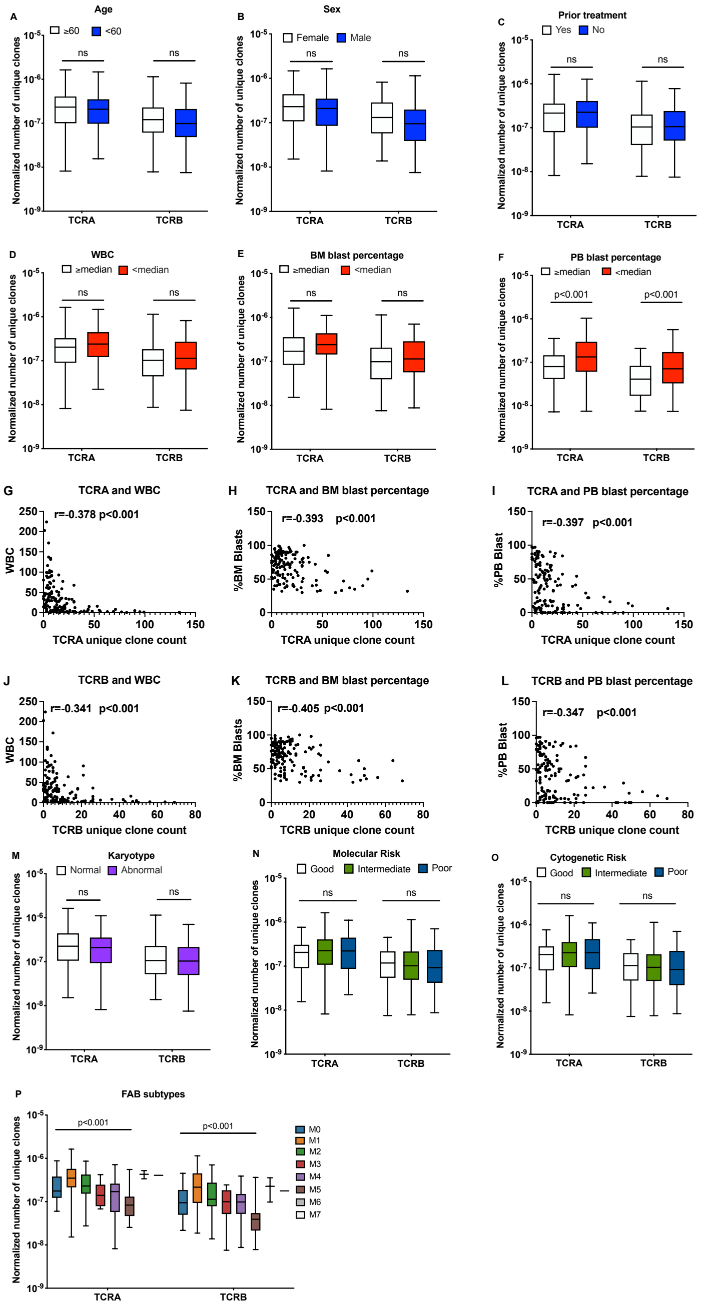

Figure S2 Normalized number of TCR unique clones is correlated with patients' clinical characteristics. Association between normalized number of unique TCR clones identified by MIXCR and age (A, TCRA: n=141, TCRB: n=139), sex (B, TCRA: n=141, TCRB: n=139), prior treatment (C, TCRA: n=141, TCRB: n=139) WBC (D, TCRA: n=141, TCRB: n=139), % BM blasts at diagnosis (E, TCRA: n=141, TCRB: n=139), % of PB blasts at diagnosis (F, TCRA: n=141, q=0.002, TCRB: n=139, q=0.002). Pearson correlation between unique TCRA clone count and WBC (G), % of BM blasts at diagnosis (H) % of PB blasts at diagnosis (I), TCRB and WBC (J) % of BM blasts at diagnosis (K), % of PB blasts at diagnosis (L). Association between normalized number of unique clones and: Karyotype (M, TCRA: n=138, TCRB: n=136), Molecular Risk (N, TCRA: n=138, TCRB: n=136), Cytogenetic Risk (O, TCRA: n=138, TCRB: n=136), FAB subtypes (P, TCRA: n=140, TCRB: n=138). Data were analyzed by unpaired t test based on log(Y) transformed data or Kruskal-Wallis with Dunn's post hoc test with p<0.05 showing significant difference between groups. FDR correction for multiple comparisons by the Benjamini-Hochberg procedure was performed and considered significant when q value is <0.05.

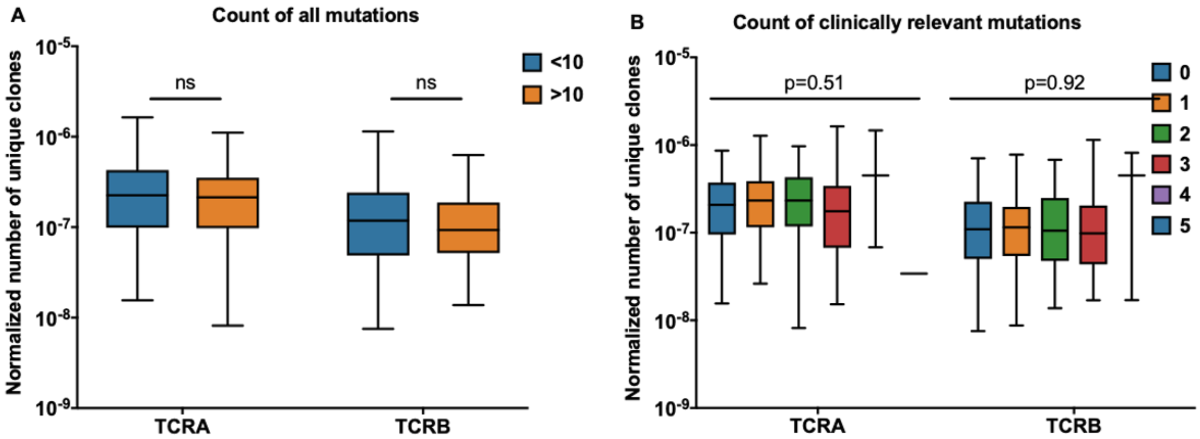

Figure S3. Association between normalized number of unique TCRA and TCRB clones identified by MIXCR and common AML mutations. Association with: a number of clinically relevant AML mutations (A), a number of all mutations (B). Data were analyzed by Kruskal Wallis test with Dunn's post-hoc test. p<0.05. showing significant difference between groups (TCRA: n=141, TCRB: n=139).

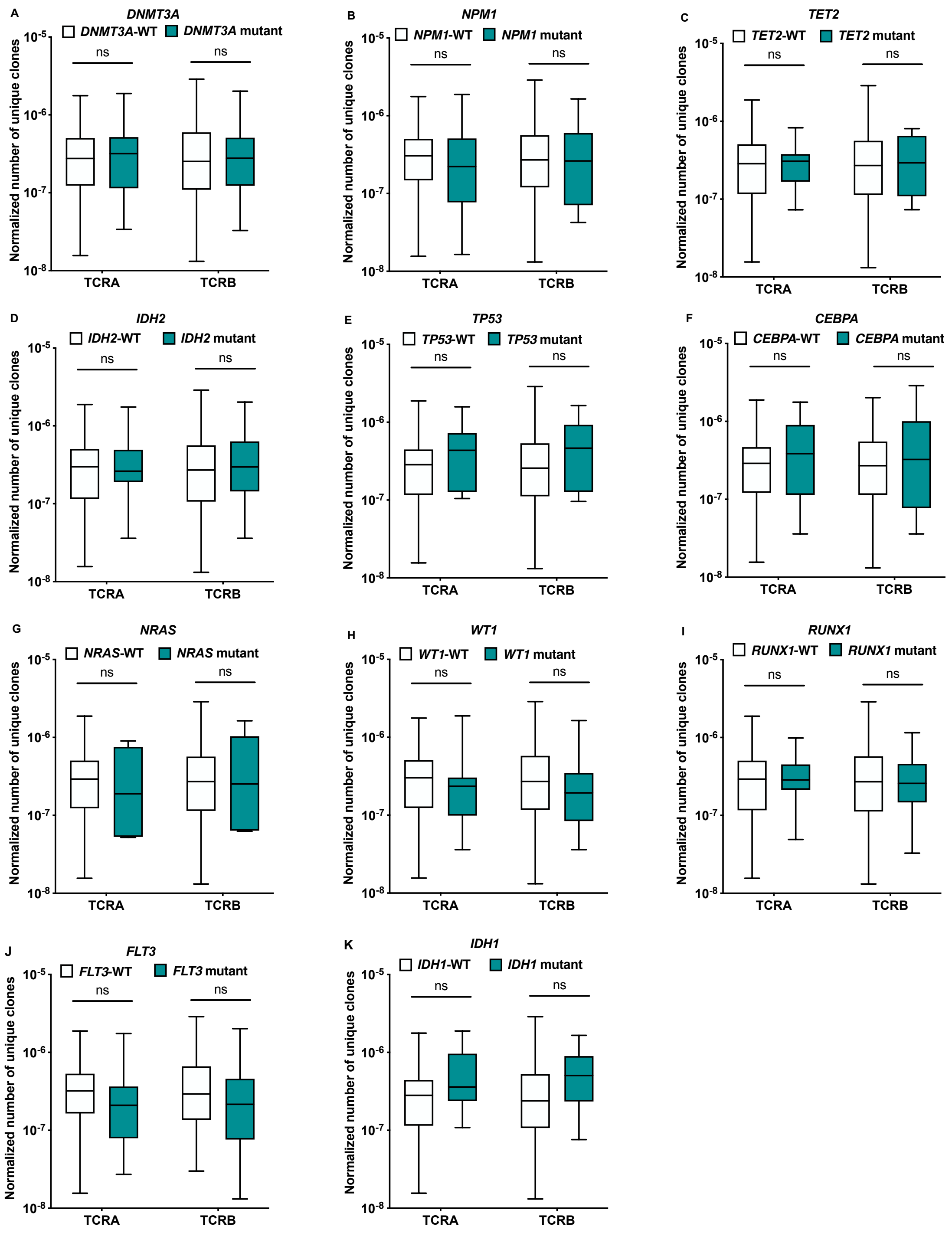

Figure S4. Association between normalized number of unique clones identified by TRUST4 and common AML mutations. Association with: *DNMT3A* (A), *NPM1* (B), *TET2* (C), *IDH2* (D), *TP53* (E), *CEBPA* (F), *NRAS* (G), *WT1* (H), *RUNX1* (I), *FLT3* (J), *IDH1* (K) . Data were analyzed by unpaired t test based on log(Y) transformed data with $p<0.05$ showing significant difference between groups (n=141). FDR correction for multiple comparisons by the Benjamini-Hochberg procedure was performed and considered significant when q value is <0.05.

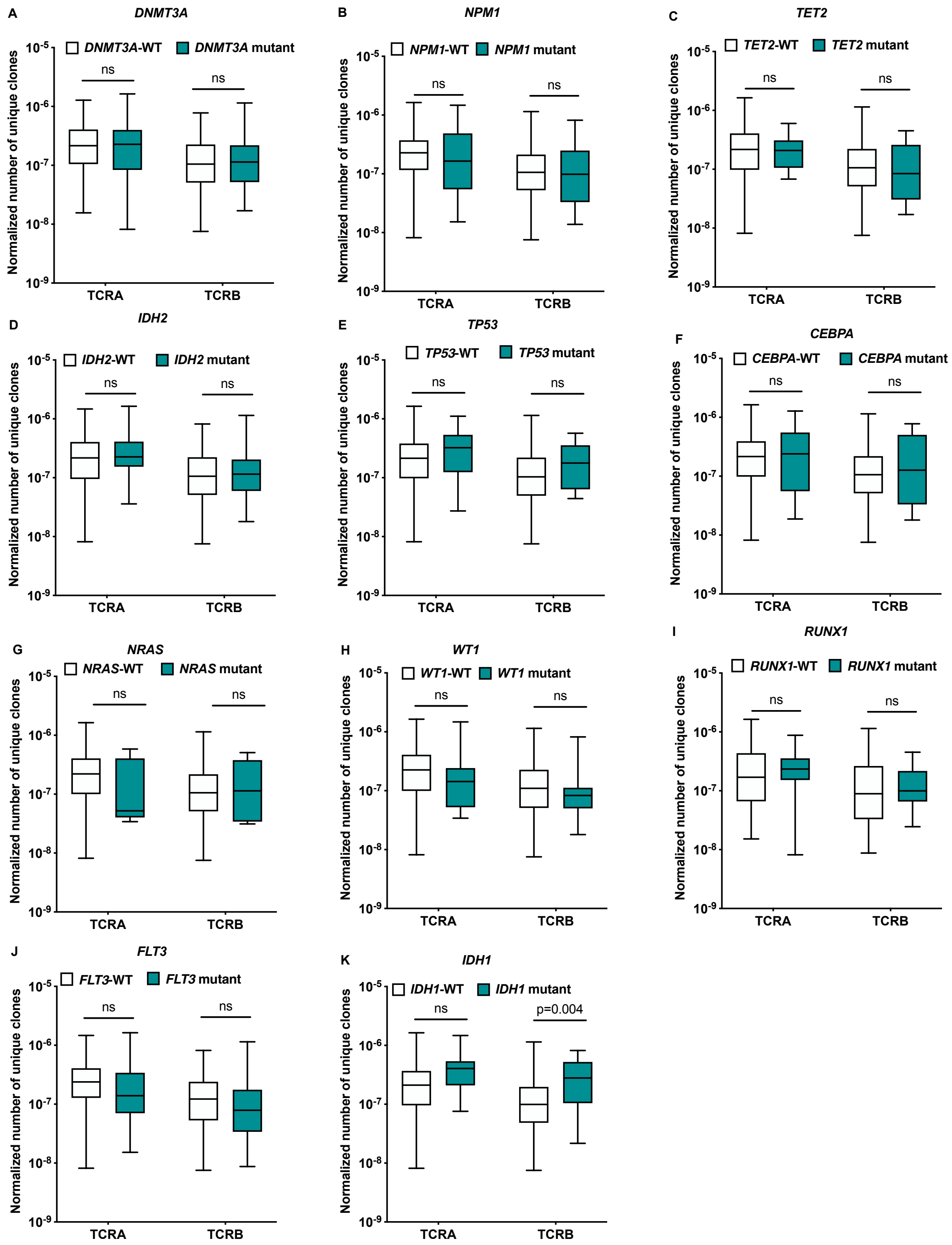

Figure S5. Association between normalized number of unique clones identified by MIXCR and common AML mutations. Association with: *DNMT3A* (A, TCRA: n=141, TCRB: n=139), *NPM1* (B, TCRA: n=141, TCRB: n=139), *TET2* (C, TCRA: n=141, TCRB: n=139), *IDH2* (D, TCRA: n=141, TCRB: n=139), *TP53* (E, TCRA: n=141, TCRB: n=139), *CEBPA* (F, TCRA: n=141, TCRB: n=139), *NRAS* (G, TCRA: n=141, TCRB: n=139), *WT1* (H, TCRA: n=141, TCRB: n=139), *RUNX1* (I, TCRA: n=143, TCRB: n=139), *FLT3* (J, TCRA: n=143, TCRB: n=139), *IDH1* (K, TCRA: n=143, TCRB: n=139, q=0.039). Data were analyzed by unpaired t test on log(Y) transformed data with p<0.05 showing significant difference between groups. FDR correction for multiple comparisons by the Benjamini-Hochberg procedure was performed and considered significant when q value is <0.05.
